# Modifying the Power and Performance of 2-Dimensional MoS_2_ Field Effect Transistors

**DOI:** 10.34133/research.0057

**Published:** 2023-03-08

**Authors:** Fulin Zhuo, Jie Wu, Binhong Li, Moyang Li, Chee Leong Tan, Zhongzhong Luo, Huabin Sun, Yong Xu, Zhihao Yu

**Affiliations:** ^1^ College of Integrated Circuit Science and Engineering, Nanjing University of Posts and Telecommunications, Nanjing 210023, China.; ^2^ Guangdong Greater Bay Area Institute of Integrated Circuit and System, Guangzhou 510535, China.; ^3^ Institute of Microelectronics, Chinese Academy of Sciences, Beijing 100029, China.; ^4^ College of Electronic and Optical Engineering and College of Flexible Electronics (Future Technology), Nanjing University of Posts and Telecommunications, Nanjing 210023, China.

## Abstract

Over the past 60 years, the semiconductor industry has been the core driver for the development of information technology, contributing to the birth of integrated circuits, Internet, artificial intelligence, and Internet of Things. Semiconductor technology has been evolving in structure and material with co-optimization of performance–power–area–cost until the state-of-the-art sub-5-nm node. Two-dimensional (2D) semiconductors are recognized by the industry and academia as a hopeful solution to break through the quantum confinement for the future technology nodes. In the recent 10 years, the key issues on 2D semiconductors regarding material, processing, and integration have been overcome in sequence, making 2D semiconductors already on the verge of application. In this paper, the evolution of transistors is reviewed by outlining the potential of 2D semiconductors as a technological option beyond the scaled metal oxide semiconductor field-effect transistors. We mainly focus on the optimization strategies of mobility (*μ*), equivalent oxide thickness (*EOT*), and contact resistance (*R_C_*), which enables high ON current (*I_on_*) with reduced driving voltage (*V_dd_*). Finally, we prospect the semiconductor technology roadmap by summarizing the technological development of 2D semiconductors over the past decade.

## Introduction

In 1965, Gordon Moore, the cofounder of Intel, extrapolated an observation and projection through historical trend that the number of transistors in a dense integrated circuit (IC) doubles about every 2 years, which has become an indestructible economic driver for the exponential growth of the semiconductor industry. It is important to note that Moore's Law [1] is not a law of nature, but a guideline that the semiconductor industry uses as a goal. In a sense, this law will fail one day as the atomic scaling approaches. The first 50 years of Moore's Law is the miniaturization of device scale, and then the optimized performance and power consumption are obtained naturally, which is the so-called “happy scaling era”. After that, the benefits of scaling down have not been enough to meet the needs for performance improvement, and the industry aimed at more extreme interconnection, mobility, and dielectric engineering, and even subverted the bulk/planar structure to obtain the performance improvement and power consumption reduction. The characteristic field effect transistor (FET) scaling length is derived as $\lambda =\sqrt{\mathrm{EOT}∙\varepsilon_{s}∙t_{s}/\varepsilon_{\mathrm{ox}}}$, where *EOT*, *ε_ox_*, *ε_s_*, and *t_s_* are the equivalent oxide thickness of gate oxide, the dielectric constant of gate oxide, the dielectric constant, and the thickness of channel materials, respectively. Therefore, transistors demand larger gate capacitances (high dielectric constant and thinner oxide thickness) and thinner channel thicknesses to maintain ideal switching characteristics at sufficiently small dimensions.

**Fig. 1.**
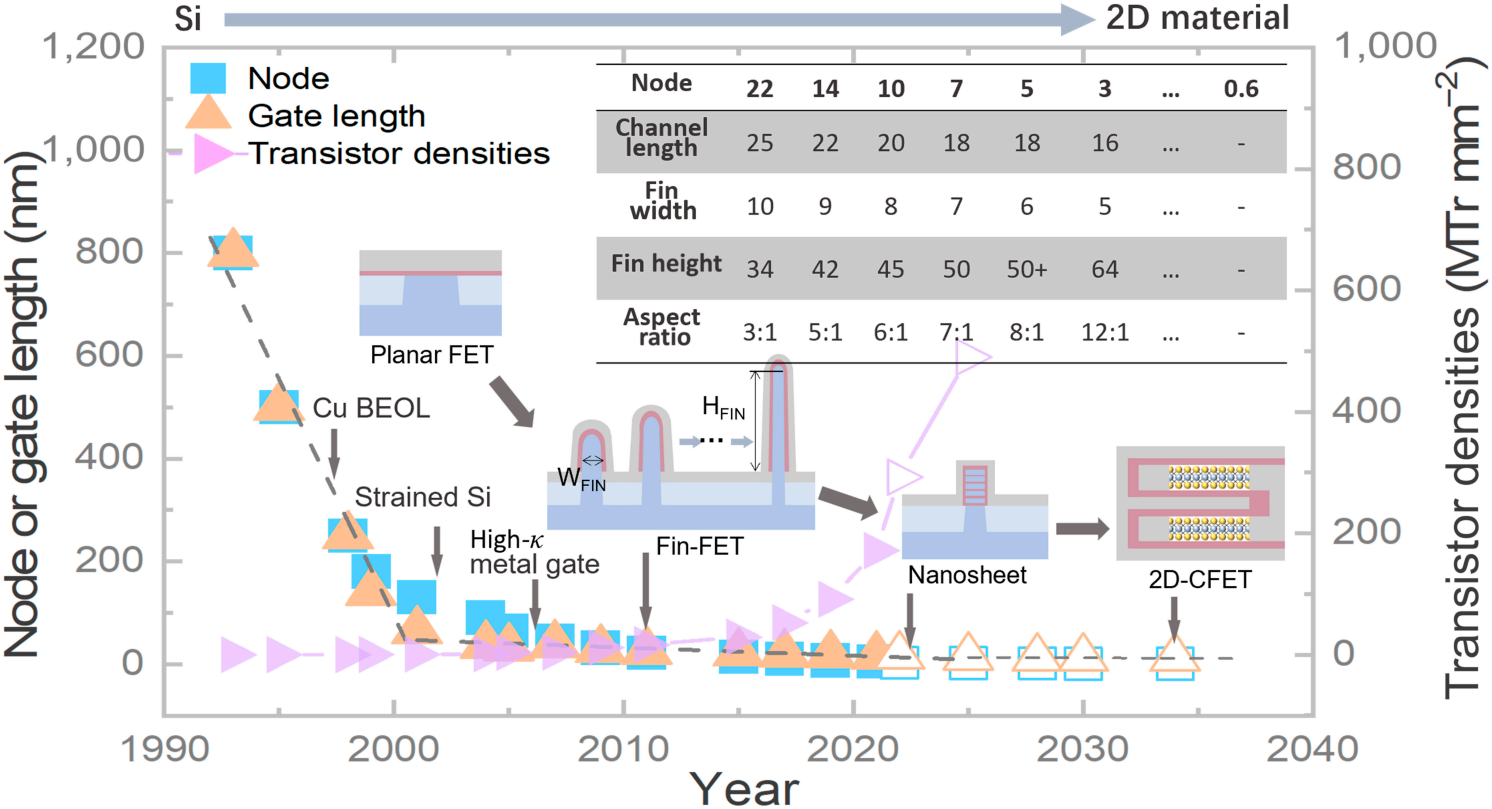
Evolution of transistors: from Si-FET to 2D-FET. The blue solid square symbol, orange solid regular triangle symbol, and purple solid triangle symbol show the relationship of node, gate length, and transistor density with year, respectively. The open symbols are the prediction data adapted from IRDS 2021 [9]. The table on the upper right gives the related parameters of Fin-FET evolution starting from 22 nm.

Figure 1 reviews the development of semiconductor technology since the 1990s. The early device characteristics roughly follow the Denard Scaling principle; that is, the channel length and the driving voltage *V_dd_* of the new-generation devices are reduced by 30% to ensure a constant electric field, which, in turn, leads to a 30% lower circuit delay *τ*. Crucially, after the 130-nm node, Dennard Scaling [2] is out of work, enabling explosive new technologies beyond scaling down. Before the 65-nm node, aluminum was replaced by copper for interconnect, resulting in lower series resistance [3]. On the other hand, SiGe increased silicon mobility by 35% through lattice strain [4,5]. During this period, the reduction of power consumption was just a windfall of the reduced *V_dd_* that comes with shrinking channel dimensions. In the following 45-nm node, the severe short-channel effect caused leakage current and degradation of the subthreshold slope ($\mathrm{SS}=\frac{\ln\left(10\right)k_{B}T}{e}\left(1+\frac{C_{s}}{C_{\mathrm{ox}}}\right)=\frac{\ln\left(10\right)k_{B}T}{e}\left(1+\frac{C_{s}+eD_{\mathrm{it}}}{C_{\mathrm{ox}}}\right)$, where *k_B_*, *T*, *e*, *C_s_*, *C_ox_*, and *D_it_* are the Boltzmann constant, the absolute temperature, the elementary charge, the areal channel capacitance, the areal gate oxide capacitance, and the density of interface traps, respectively). In fact, the industry has employed oxynitride instead of pure SiO_2_ as a gate dielectric since the 1990s to raise the dielectric constant and to offer other advantages against dopant diffusion. By the 65-nm node, the thickness of oxynitride has been reduced to 1.2 nm [6], which approaches the quantum tunneling limit, and the next-generation devices (45 nm node) have to introduce a higher dielectric constant (high-*κ* metal gate abbreviated as HKMG) to enhance gate modulation while suppressing tunneling current [7].

An even bigger technological leap occurred at 22 nm, and since then, semiconductor devices embraced the biggest structural revolution since complementary metal oxide semiconductor, with Fin-FET and ultrathin body silicon on insulator (UTB-SOI) replacing the planar devices and bulk silicon technologies, respectively [8]. For more advanced nodes, SOI requires a wafer-scale fully depleted nanosheet with a thickness of sub-3 nm, which is a huge challenge to the present technology. As shown in the inset of Fig. 1, Fin-FET has undergone sixth-generation testing since its commercial application, up to the state-of-the-art 3-nm node. It is worth noting that, to obtain more effective gate control, the Fin thickness is continually decreased with increasing gate height to compensate for current decline. For the 3-nm node, the aspect ratio exceeds 12:1 (64 nm vs. 5 nm) [9], while maintaining a smooth interface. Manufacturing such an aspect ratio extremely challenges both cost and processing, and therefore, gate-all-around (GAA)-FET is proposed to enhance gate control, thanks to the more comprehensive gate coverage and thinner channel compared to Fin-FETs. Remarkable performance and *SS* improvements have been achieved by vertically stacking multibridge channel (MBC) FET [8].

For device structure, both Fin-FET and MBC-FET are approaching quasi-2-dimensional (2D) nanosheets, and a better solution is the thinner semiconductor layer. 2D semiconductors, especially transition metal dichalcogenides (TMDs) with a general chemical formula of MX_2_, exhibit atomically thin thickness and provide perfect gate modulation on an extremely scaled planar or MBC-FET structure [10–21]. TMD materials also show tunable bandgap and carrier mobility by engineering the element composition [22–24], layer number [25,26], and strain [26,27], which are promising for high-performance transistors at sub-3-nm nodes. Moreover, the growth of TMD single crystal over wafer was realized, which is the key step toward commercialization [28,29]. Therefore, with the proper combination of materials and structures, TMD and their heterojunctions will enable numerous new electronic and optoelectronic applications [30–32].

In this paper, we will review the development of TMD logic devices, especially the key technologies to achieve high-performance, low-power transistors through systematic optimizations of mobility, dielectric, and contact. First, we expound the characteristics of ideal 2D metal oxide semiconductor field-effect transistors. Next, we start with key device metrics and summarize the most important progresses published in the literature. Finally, we discuss the optimal device performance data reported to date and prospect the requirements for future industrial applications.

## The Ideal 2D Transistor

**Fig. 2.**
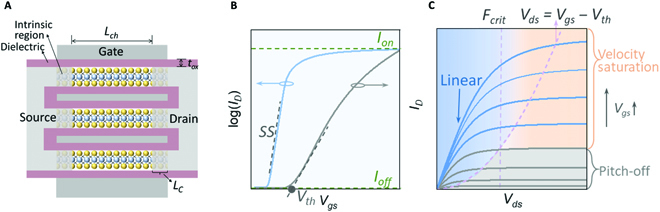
Ideal 2D MBC-FET and its electrical characteristics. (A) Structure of a typical 2D MBC-FET. The *L_C_*, *L_ch_*, and *t_ox_* are the contact length, the effective channel length, and the thickness of the gate dielectric, respectively. (B and C) Transfer (*I_D_ − V_gs_*) and output (*I_D_ − V_ds_*) characteristics of a typical n-type 2D-FET. The *SS*, *V_th_*, *I_on_*, *I_off_*, and *F_crit_* are the subthreshold swing, the threshold voltage extracted in the linear region, the on-state and off-state current, and the critical field, respectively.

Benefiting from van der Waals (vdW) stacking of 2D semiconductors, it is more feasible to realize MBC-FET and complementary FET (CFET) structures compared to bulk materials, and thus to obtain superior device characteristics. Figure 2A schematically illustrates the typical 2D MBC-FET, consisting of multiple vertically stacked 2D semiconductor channels. Paralleling multiple channels provides a larger drive current while saving footprint on wafer. The total resistance arises mainly from the contact resistance (*R_C_*) and the channel resistance (*R_ch_*). The latter can be expressed as:

$$R_{\mathrm{tot}}=\left(R_{C}+\frac{L}{WC_{\mathrm{ox}}\mu \left(V_{\mathrm{gs}}-V_{\mathrm{ds}}\right)}\right)/N$$ (1)

where *L*, *W*, *N*, *C_ox_*, *μ*, *V_gs_*, and *V_ds_* are the effective channel length and width, the stacking layer number, the areal capacitance of the gate oxide, the mobility, the gate voltage, and the drain voltage, respectively. Therefore, for a specific node, lower *R_c_*, higher mobility, and greater areal capacitance are critical for better drive capability. Figure 2B and C plots the representative transfer and output curves, where some key metrics are marked beside. Note that, small *SS* and low off-state current are essential for low power consumption (dynamic and static). On the other hand, a large on-state current is also highly desired, as seen in both the output and transfer curves, which points to good contact and high mobility. Furthermore, a well-controlled threshold voltage (*V_th_*) that can serve as an indicator of process variation is especially important for large-scale integration. Next, we will systematically discuss the principal performance metrics at the device level.

## Scattering and Mobility Booster

Carrier mobility represents the drift velocity per unit electric field, and is a key parameter to characterize the charge transport. Mobility can be expressed as $\mu =\frac{e\tau_{m}}{m*}$, closely related to the mean free time (*τ_m_*) and the effective mass (*m*^∗^). For a specific semiconductor, scattering determines *τ_m_* and thus its mobility. Compared with that in Si, the charge transport in TMD is more susceptible to scattering (intrinsic and extrinsic). According to Matthiessen's Law, in a TMD containing multiple scattering mechanisms, the free carrier mobility can be evaluated by [33,34]

$$\mu {\left(T\right)}^{-1}=\mu_{\mathrm{ph}}{\left(T\right)}^{-1}+\mu_{\mathrm{SO}}{\left(T\right)}^{-1}+\mu_{\mathrm{CI}}{\left(T\right)}^{-1}+{\mu_{\mathrm{DE}}}^{-1}$$ (2)

where *μ_ph_*, *μ_SO_*, *μ_CI_*, and *μ_DE_* are the mobilities associated with the intrinsic phonons, the surface optical phonons (SOs), the Coulomb impurities (CIs), and the defects, respectively. Therefore, quantitatively determining the contributions of the various scatterings to the resistivity poses an enormous challenge, which invokes collaboration between the experimental study with precisely controlled parameters and the numerical modeling so as to interpret the relevant mobility variations with scattering.

**Fig. 3.**
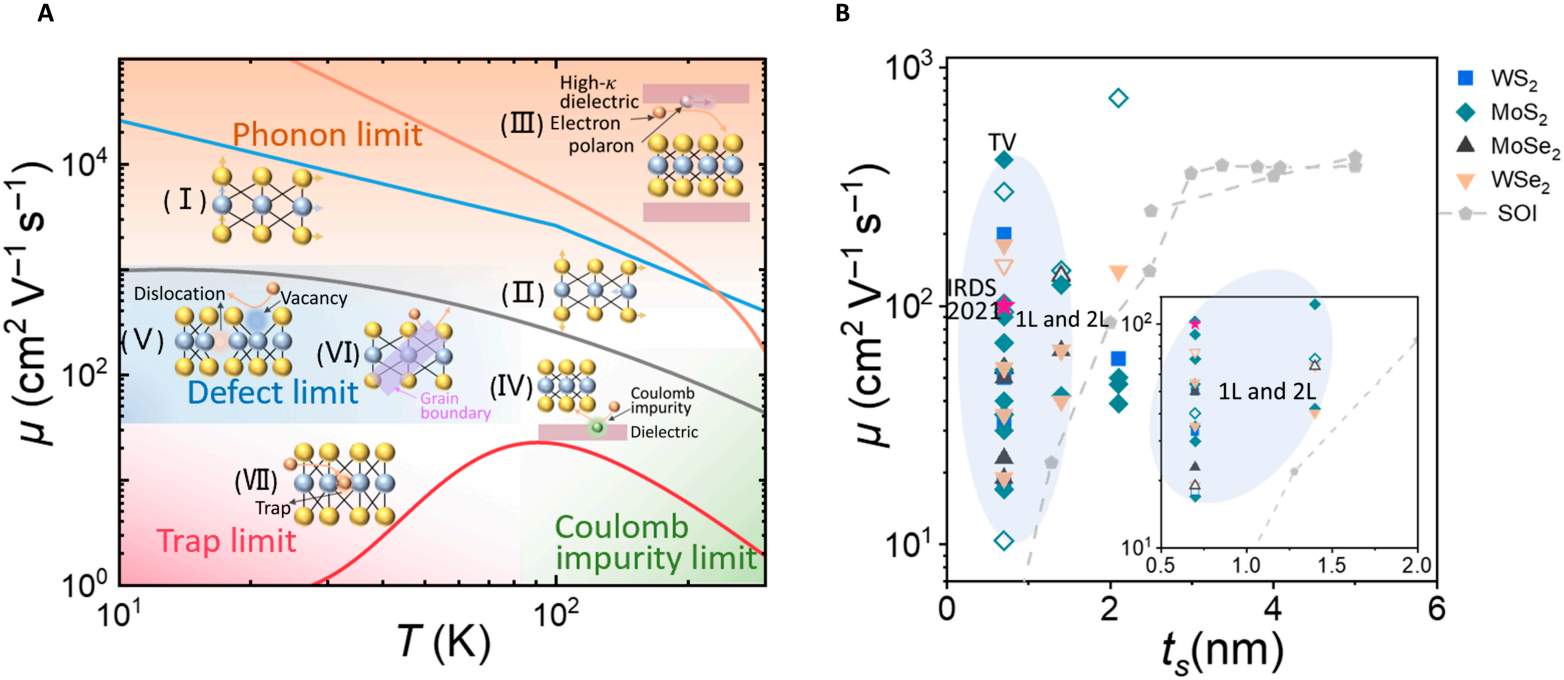
Scattering and mobility in 2D-FETs. (A) Mobility as a function of *T* for different scatterings in a typical MoS_2_ transistor. The orange, blue, and gray lines represent mobilities limited by the SO scattering, the intrinsic phonon scattering, and the Coulomb impurity scattering in monolayer MoS_2_, respectively_._ The red line reveals mobility degeneration after introducing the effect of localized states and traps. (I) Deformation potential of phonons caused by atomic vibrations. (II) Frohlich interaction caused by polar optical phonons. (III) Surface phonon scattering. (IV) Coulomb impurity scattering. (V) Atomic defect scattering. (VI) Defect and grain boundary scattering. (VII) Trap effects. All halos in schematics represent potential. (B) Mobility versus the channel film thickness for CVD-growth TMD (solid symbols), mechanically exfoliated TMD (hollow symbols), and ultrathin silicon-on-insulator. The oval region highlights the mobility of monolayer and bilayer TMD. TV is the abbreviation of the theoretical value, for monolayer MoS_2_. The inset shows more details of the sub-2-nm region. The mobility values of MoS_2_, WSe_2_, MoSe_2_, WS_2_, and SOI are reproduced from Refs. [14, 29, 32, 49–53, 56, 57, 60–62, 67, 69, 70, 72, 73, 127, 128, 148–151], Refs. [110, 152–155], Refs. [156–158], Refs. [110, 129, 159], and Refs. [160–164], respectively.

Take MoS_2_ as an example, the *μ* − *T*^−1^ relationship under different scattering mechanisms is qualitatively plotted in Fig. 3A. The highest or theoretical mobility of ~410 cm^2^ V^−1^ s^−1^ for monolayer MoS_2_ at room temperature should signify a transport mode dominated by the intrinsic phonon scattering, which consists of longitudinal acoustic (Fig. 3A (I)) and optical (Fig. 3A (II)) wave phonons. Acoustic phonon scattering contributes mainly at low temperatures less than 100 K and its scattering rate linearly depends on the temperature (*T*), and thus the intrinsic phonon-limited mobility varies as *μ* ∝ *T*^−1^. When the temperature is greater than 100 K, the optical wave phonons become the major contributor, and then the intrinsic phonon-limited mobility varies as *μ* ∝ *T*^−1.69^ [35]. Apart from the intrinsic electron-phonon scattering, remote interaction with the polar optical phonons at the dielectric surface (Fig. 3A (III)), named SO scattering, gives rise to even stronger temperature dependence, in particular at high temperatures [36]. SO scattering is proportional to the ionization strength of the chemical bonds in dielectric, and the molybdenum-oxygen (Mo-O) bonds of oxide with high dielectric constants have stronger ionization strengths, which can lead to more severe SO scattering [37]. Differing from that in traditional Si transistors, SO scattering could significantly degrade the mobility of 2D-FETs, especially for GAA devices with double-side high-*κ* dielectric [36]. Under the combined influence of intrinsic phonons and SO, the room-temperature mobility of monolayer MoS_2_ FETs using high-*κ* dielectric may be as low as 200 cm^2^ V^−1^ s^−1^ [38,39]. Even so, the experimental mobility of monolayer MoS_2_ could be much lower than the theoretical value. The nature of the charge transport in MoS_2_, however, remains poorly understood despite the extensive theoretical and experimental studies performed to date. In the early days, the mobility of monolayer MoS_2_ was around 10 cm^2^ V^−1^ s^−1^, and the *μ* − *T*^−1^ relationship exhibited obvious insulating behaviors [40–42] (Fig. 3A (VII)). Combining the electrical transport measurements and the atomic-resolution transmission electron microscopy, Qiu et al. found a large number of intrinsic sulfur vacancies presented in the MoS_2_ lattice. These vacancies acted as localized states, giving rise to hopping transport at low carrier concentration (*n*_2*D*_) [43]. Besides, a particularly important source of scattering stems from the CIs at the semiconductor/dielectric interface, which are believed to be the limiting factor of the present MoS_2_ devices [44]. The intensity of CI scattering depends on the CI density. The essence of CI scattering is the interaction with the electric field, which can be screened by the external field. Accordingly, the screened potential reads [45,46] $\phi_{q}^{\mathrm{scr}}=\frac{\phi_{q}}{\varepsilon_{2D}\left(q,T\right)}$, where $\phi_{q}=\frac{e^{2}}{\left(\varepsilon_{\mathrm{box}}+\varepsilon_{0}\right)q}$ and $\varepsilon_{2D}\left(q,T\right)=1+\frac{2\varepsilon_{\mathrm{el}}\left(q\right)}{\varepsilon_{\mathrm{box}}+\varepsilon_{0}}$ are the bare potential and the screening effect by the substrate and the free electrons, respectively; *ε_box_* and *ε*_0_ are the permittivity of the substrate and vacuum; *ε_el_*(*q*) corresponds to the electronic part of the dielectric function and depends on the carrier density *n*. As *n* and *ε_box_* increase, screening gets stronger, reducing the scattering potential and thus increasing the CI-limited mobility.

To obtain higher mobility, numerous interface and defect engineering designs have been proposed for TMD. Yu et al. [34] developed thiol chemistry to achieve in situ defect modification, which effectively passivates defects and traps, significantly increasing mobility at room temperature. Furthermore, they introduced the screening effect through a high-*κ* with high carrier concentration to suppress CI scattering and realized a phonon-limited mobility of ~150 cm^2^ V^−1^ s^−1^ at room temperature in monolayer MoS_2_ [33]. It was demonstrated that by sandwiching a monolayer MoS_2_ channel in-between boron nitride (BN) layers, CI scattering can be considerably suppressed, leading to high mobility at room temperature and at low temperatures [41,47,48]. Moreover, optimized chemical vapor deposition (CVD), especially single-crystal growth, is an effective and industry-compatible technique to reduce defects and increase mobility [29,30,32,49–73]. Currently, for most 2D semiconductors, CVD films exhibit properties that far exceed those of mechanically exfoliated flakes.

Figure 3B summarizes the thickness-dependent mobility of Si and 2D semiconductors, including MoS_2_, MoSe_2_, WS_2_, and WSe_2_. The conventional Si is subject to the repeated thinning and etching processes, and its mobility drops sharply as the SOI thickness reduces below 3 nm. This will severely restrict the device performance at the advanced nodes. By contrast, the thickness of monolayer TMDs is only 0.7 nm. Although the experimental mobility is far lower than the theoretical prediction, it still holds distinct advantage against SOI to provide one-decade higher mobility at similar thickness. In future, an ultraclean, nondestructive manufacturing environment and the delicate design of device structures are vital to the development of 2D-FETs.

## Dielectric Integration and Interface Engineering

**Fig. 4.**
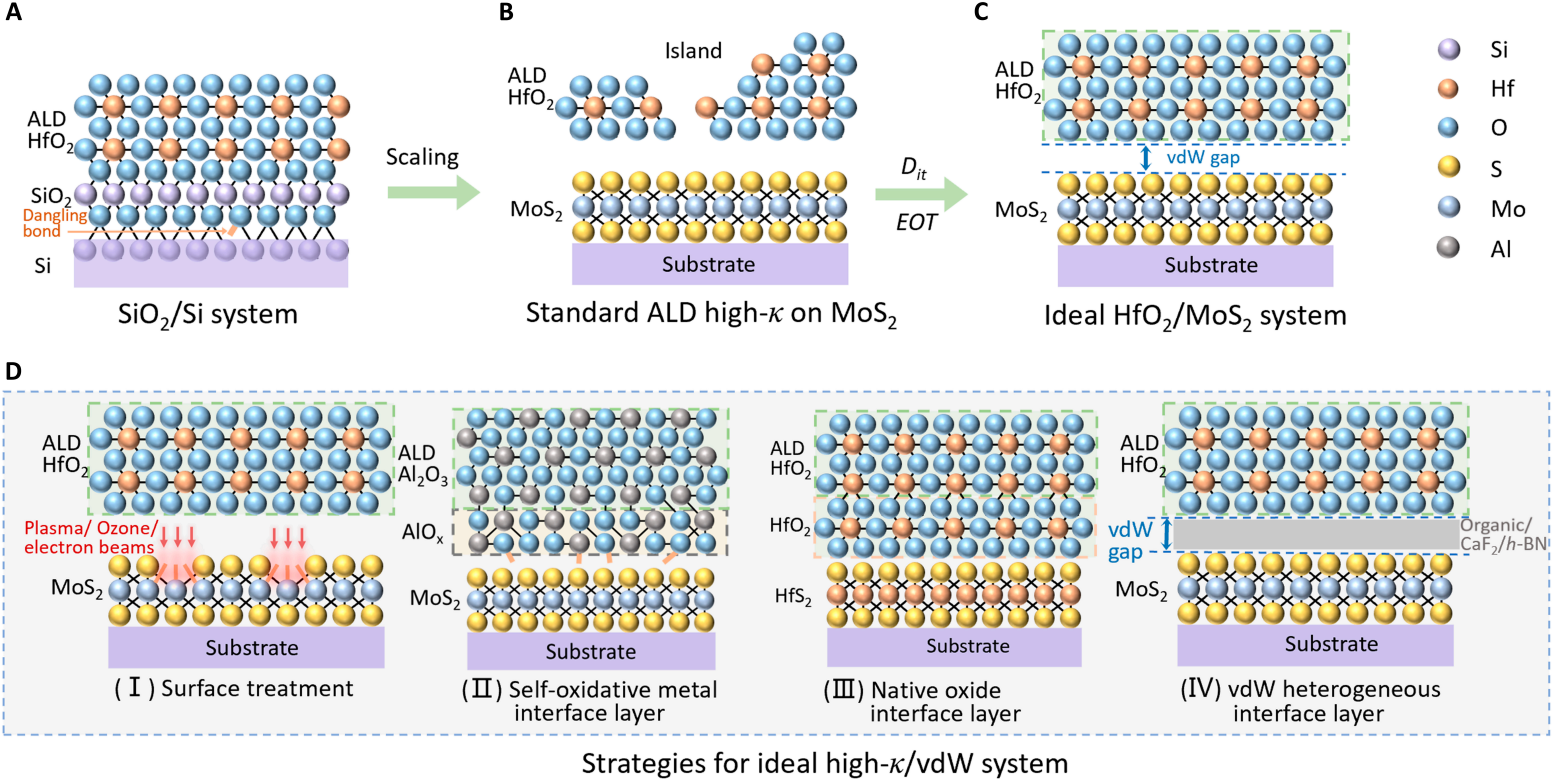
Dielectric engineering in 2D-FETs. (A) Schematic of high-quality SiO_2_/Si interface (*D*_it_ < 10^10^ cm^−2^ eV^−1^) thanks to a tight covalent bond. (B) Schematic of direct ALD high-*κ* dielectric on 2D channel to continue Moore's Law such as HfO_2_/MoS_2_. (C) Schematic of an ideal dielectric/2D semiconductor system. (D) Schematic illustration of 4 strategies for integrating high-*κ* dielectric onto 2D channels, where (I) is for surface functionalization strategy, (II) is for oxidation of the metal seed layer above the channel, (III) is for channel self-oxidation, and (IV) is for vdW integration strategy by inserting an intermediate buffer layer between the 2D channel and high-*κ* dielectric layer, such as organic/CaF_2_/*h*-BN.

Scaling down has long relied on dielectric thinning for greater gate controllability (small *SS* and high transconductance) and for lower leakage. As native oxide, SiO_2_ benefits from the nearly perfect interface with Si and has transformed from an ideal dielectric layer to an ideal interface layer in the high-*κ* era (Figure 4A) [74,75] with *D_it_* below 10^10^ cm^−2^ eV^−1^ at the SiO_2_/Si interface [76] and an *EOT* of 0.6 nm for the state-of-the-art technology node [77]. Nowadays, atomic layer deposition (ALD) can produce very precise ultrathin high-*κ* dielectric with SiO_2_ as a buffer layer. The layer-by-layer deposition of ALD depends on the precursor adsorption and nucleation by interfacial dangling bonds. Therefore, the surface rich in dangling bonds is the key to achieving a high-quality ALD dielectric. For 2D materials, however, the inherent absence of out-of-plane dangling bonds, responsible for vdW stacking, poses a huge obstacle to ALD-based dielectric integration. When dielectric is directly deposited on 2D materials by ALD, the precursors preferentially nucleate at the sites of lattice defects to form island-like growth, which exacerbates interface properties (Figure 4B). In fact, the ideal integration of dielectric for 2D devices should be through direct vdW stacking of high-*κ* dielectric and 2D materials (Fig. 4C). However, these methods are neither compatible with industrial technology nor applicable to top-gate architecture. Therefore, dielectric integration in manufacturing top-gate 2D-FETs must involve interfacial modifications.

In the past decade, several strategies have been developed for interfacial modification of 2D materials, especially for 2D semiconductors (Fig. 4D): (I) surface treatment, (II) self-oxidative metal interface layer, (III) native oxide interface layer, and (IV) vdW heterogeneous interface layer. Regardless of the method utilized, the effective *EOT* will be the sum of the *EOT* of interface layer and ALD oxide. Thus, the optimum interface layer should exhibit the lowest *EOT* while maintaining the extremely low *D_it_*.

The purpose of surface treatment is to create uniform point defects and dangling-bonds on 2D surface, which assist the adsorption and the nucleation of ALD precursors. Typical treatment methods include plasma [78–80], ozone [81,82], and electron beam irradiation [83,84]. Obviously, surface treatment sacrifices interface quality and will sizably raise *D_it_*. Furthermore, introduction of these interfacial defects further increases the source of CI scattering, resulting in irreversible degradation of mobility. Huang et al. [78] achieved ~1.5 nm of Al_2_O_3_ through water plasma treatment and the corresponding *D_it_* reached 2.1 × 10^12^ cm^−2^ eV^−1^, which is more than 2 orders of magnitude higher than that at SiO_2_/Si [76]. Another widely used interface layer aims to evaporate a thin chemically active metal (such as aluminum [85–87], magnesium [85], and yttrium [85,88,89]). The self-oxidative product can serve as a dielectric or ALD interface layer. This method is also commonly applicable to other systems such as carbon nanotube [90] and organic transistors [91]. The self-oxidative metal methodology has good versatility for different materials. The resultant interface layer maintains a relatively high dielectric constant, but inevitably incurs channel damage during the evaporation process [92]. The fatal drawback is that, in order to ensure the interface uniformity and controllable leakage, the thickness of the accompanying self-oxidative layer usually exceeds a few nanometers, which is difficult to meet the *EOT* requirements of advanced node. As a result of combining the advantages of the aforementioned methods, a more advanced interface layer—2D native oxide—was invented recently. Typical examples are HfS_2_ [93] and Bi_2_O_2_Se [94]. Lai et al. [93] fabricated top-gate FET with a low *D_it_* of 6 × 10^11^ cm^−2^ eV^−1^ between HfS_2_ channel and converted HfO_2_ dielectric. Li et al. [94] achieved 0.9 nm of *EOT* dielectric layers of Bi_2_SeO_5_ through layer-by-layer oxidation of the underlying 2D Bi_2_O_2_Se semiconductor. The corresponding leakage current was lower than 1 × 10^−7^ A·cm^−2^ under an external field strength of 1 MV·cm^−1^. This method is expected to meet both of the requirements for interface quality and *EOT* under precise control. However, the problem is also obvious: only a few specific 2D semiconductors can form dense native oxide, especially Mo-based and W-based TMD with the greatest application potential, for which this methodology is completely infeasible. Hence, another strategy, viz., vdW heterogeneous interface layer, is more friendly to 2D materials owing to ultrasmooth interface and damage-free vdW interaction. Wang et al. [95] used organic molecule perylene tetracarboxylic acid (PTCA) to treat graphene and realized noncovalent functionalization, assisting the deposition of high-quality ALD dielectric. Afterwards, Li et al. attained an organic crystal interface monolayer of only 0.3 nm by choosing new organic molecules of perylenetetracarboxylic dianhydride (PTCDA) in conjunction of optimizing the molecular deposition process. Breaking through of 1 nm of *EOT* was made for 2D materials [96]. In further statistical studies, Yu et al. [97] proved that the PTCDA interface layer could enable high dielectric reliability comparable to that of SiO_2_/Si. The noncovalent monolayer of organic crystal has excellent interface quality, versatility, and reliability for 2D devices, but is restricted by the intrinsic low dielectric constant. Therefore, it is still very challenging to achieve a more aggressive *EOT* of sub-1 nm by means of this technique. More recently, Liu et al. employed a 2D inorganic molecular crystal of Sb_2_O_3_ as an ALD interface layer. Benefiting from its ultrahigh dielectric constant of 11.5 and the equally perfect interfacial quality to PTCDA, inorganic molecular crystals demonstrated great potential to become a new candidate of interfacial layer [98].

In addition to the traditional ALD oxide, 2D layered dielectrics have also been considered to be a unique solution for their 2D vdW properties. The representative, first-generation layered dielectric should be hexagonal BN (*h*-BN). Yet, its dielectric constant is only 4 [99]. Moreover, the serious leakage current [100] and the layer-by-layer transfer make *h*-BN almost impossible to be applied for larger-scale integration. A new generation of layered dielectric has been reported in recent years. For example, layered calcium fluoride [101,102] and strontium titanate [103] permitted vdW integration, realizing a record *EOT* down to 0.6 nm with excellent interface between high-*κ* and 2D semiconductors. However, such technologies are still in demonstration for back-gate devices. Future development of top-gate integration will be essential for CFETs and MBC-FETs.

In principle, *EOT*, gate leakage currents (*I_g_*), and *D_it_* of high-quality dielectric/semiconductor systems must be strictly controlled to be lower than 1 nm, 10^−2^ A·cm^−2^ [104], and 10^10^ cm^−2^ eV^−1^ [96,105], respectively. The quality of the 2D channel and the gate dielectric layer, and the breakdown voltage are critical for top-gate or GAA 2D-FET as well. Currently, vdW integration (as shown in Fig. 4D (IV)) is a promising solution to the damage-free manufacturing of high-*κ* dielectrics onto 2D semiconductors, while satisfying the needs of *EOT*, *I_g_*, and *D_it_*. Of course, the premise is to find a high-*κ* 2D dielectric material suitable for 2D semiconductors.

## Ohmic Contact for 2D-FET

**Fig. 5.**
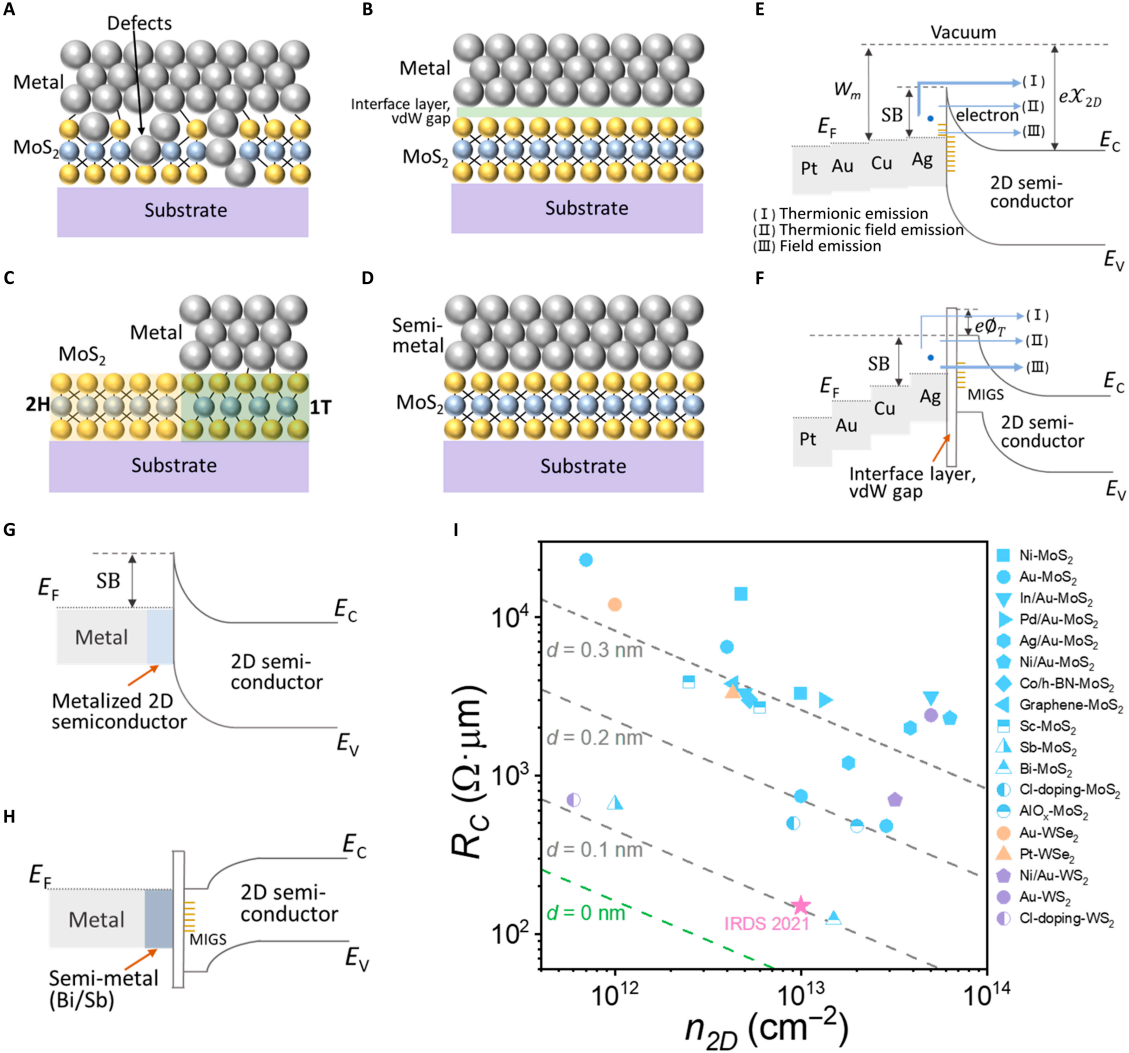
Contact engineering in 2D-FETs. (A to D) Schematics of 4 kinds of contact strategies. (A) Conventional metal deposition accompanied by a lot of defects generated on MoS_2_ surface. (B) Metal–insulator–semiconductor contact with a vdW gap between metal and MoS_2_. (C) Metallic phase 2D materials contact. (D) Semimetal/MoS_2_ contact. (E to H) Energy band diagram of different metal–2D semiconductor contacts, corresponding to the strategies of (A) to (D), respectively. There are 3 different injection mechanisms in (E) and (F), including thermionic emission (I), thermionic field emission (II), and field emission (tunneling) (III). In (E), the thermionic emission dominates. In (F), the tunneling current dominates. The yellow, dense short lines represent FLP. *E*_F_, *E*_C_, and *E*_V_ represent the Fermi level of the metal, and the conduction and valence bands of the 2D semiconductor, respectively. (I) Benchmark *R_C_* for monolayer 2D semiconductors with different contacts. The dashed lines exemplify the theoretical *R_c_* at different thicknesses of the vdW gap (*d*). The green dashed line (*d* = 0 nm) denotes the quantum limit for *R_c_* approached by mature semiconductor technologies. The *R_c_* values of WSe2, MoS2, and WS2 are reproduced from Refs. [146, 165], Refs. [110, 121, 125, 129, 133, 134, 147, 148, 166–173], and Refs. [129, 147, 174], respectively.

At the advanced nodes, the physical channel length is only of dozen nanometers, and (quasi-)ballistic transport within mean free path dominates [106]. Consequently, the influence of mobility on the on-state current of metal oxide semiconductor field-effect transistors greatly attenuates. For ultrashort channel, *R_C_* contributes mostly to the total resistance [107]. The Schottky barrier (SB), which is the energetic difference between the valence (or conduction) band edge of the semiconductor and the work function (WF) of the contact metal, blocks carrier injection from the source into the semiconductor and becomes the most important origin of *R_C_*. According to the Schottky–Mott limit, the SB height of metal–semiconductor (M–S) contact can be manipulated by adjusting metal’s WF. However, SB height is usually insensitive to WF. In reality, the Fermi level is often pinned by interface states, known as Bardeen limit [108,109]. Fermi level pinning (FLP) influences charge injection from metal into semiconductor at 2 levels: (a) metal-induced gap states (MIGS), which are ubiquitous in M–S contact [110–113] and (b) surface states resulting from contact interface traps or defects. FLP effect presents in almost all M–S junctions and has never been resolved. Yet, in commercial devices, this issue can be effectively circumvented by using degenerate doping to form an ultrathin tunneling barrier [114–116]. Owing to the atomic-scale thickness and the doping-free nature, SB in 2D-FET is significantly dictated by the contact characteristics. Take the conventional evaporated metal on MoS_2_ as example (see Fig. 5A and E for details), the intrinsic and extrinsic defects (created by high-energy metal atoms and clusters bombarding during metal deposition) introduce large density of states, which, in turn, pins the Fermi level near the midgap states [92,117–120]. In this case, no matter how the metal’s WF changes, the carriers will always experience a similar injection barrier, manifesting almost the same transport behavior [121–126].

Therefore, in order to satisfactorily obtain *R_c_*, 2 strategies can be utilized: (a) contact doping to make metallic contact or tunneling barrier and (2) MIGS suppression to minimize the SB height. The mainstream strategies for *R_C_* optimization are summarized in Fig. 5I. Inherited from silicon technology, contact doping is a consequential approach to reducing injection barrier and thus *R_C_* for whatever n-type and p-type devices [127–134]. Yang et al. [129] reported that, by employing chloride molecular doping to reduce SB width, *R_C_* of few-layer WS_2_ and MoS_2_ were decreased to 0.7 kΩ·μm and 0.5 kΩ·μm, respectively. Gao et al. [130] achieved degenerate p-type doping and observed hole transport in the MoS_2_ channel for the first time, where the specified MoS_2_ was treated by electronegative materials such as MoO_3_ or MoO_2_. Metallic phase 2D materials are another effective strategy [135–138]. The metallic 1T phase of MoS_2_ can be locally induced by alkali metal intercalation on semiconducting 2H phase, thus decreasing *R_c_* to 200 to 300 Ω·μm at zero gate voltage [135]. Also, the metallic 1T phase of vdW materials can be locally induced by strain [136], laser beam irradiation [137], and argon plasma bombardment [138] on semiconducting 2H phase. Recently, Wu et al. attained *R_C_* as low as 250 Ω·μm through atomically ultraclean vdW vanadium diselenide contacts [139]. A schematic of the energy band diagram and the structure of phase-engineered contacts are provided in Fig. 5C and G, respectively. For 2D semiconductors, however, the metallic phase is a thermodynamically metastable structure, which causes serious reliability and stability issues to the potential electronic applications. The vdW interaction of 2D semiconductors is thus expected to fundamentally eliminate the FLP effect so as to achieve ohmic contacts, as told by the Schottky–Mott limit. Yet, in the early works, the tunneling interface layer was the mainstream solution to reduce defects caused by evaporation. Researchers employed ALD-deposited oxide or transferred *h*-BN intercalated interfaces to form metal–insulator–semiconductor contacts, effectively reducing the SB height to be around 35 meV (see Fig. 5B) [122,140–143]. In addition, the graphene interface layer exhibits better conductivity and tunable Fermi level, offering more possibilities to new contact structures [144,145]. Despite that, the interface layer widens the vertical tunnel barrier (*e*∅*_T_*) between metal and 2D semiconductor (see Fig. 5F), raising *R_C_* again. On this basis, Liu et al. proposed a laminating metal contact with weak dipole polymer as channel capping layer to create substantially disorder-free interface. The SB height was dictated by the metal’s WF and thus highly tunable, approaching the Schottky–Mott limit [92]. Other researches also showed that deposition of indium and platinum at low temperature and with low diffusion energy can produce ultraclean vdW interface, which led to *R_C_* as low as 0.8 kΩ·μm and 3.3 kΩ·μm, for n-type and p-type transistors, respectively [146,147]. Compared with the transferred electrodes, this method is more industrially compatible. Recently, Shen et al. [110] reported a new approach to make ohmic contacts for monolayer MoS_2_ by using semimetallic bismuth (Bi), where MIGS were considerably suppressed and degenerate states were spontaneously generated in the TMD (Fig. 5D and H). As a result, they achieved zero SB height and a record-low *R_C_* of 123 Ω·μm, which paved a new way toward ultralow *R_C_* for 2D ohmic contact. Following that, a new semimetal contact of stibium (Sb) was discovered by Intel, TSMC, etc. [148], which showed similar contact characteristics to Bi but with better stability and reliability.

Finally, one can explore what are the critical factors of *R_C_* in a vdW system. Theoretically, the quantum limit of *R_C_* can be expressed by [149]

$$R_{c,\min}W=\left(\frac{h}{2e^{2}}\right)e^{\frac{4\pi d\sqrt{m*\left(W_{m}+\chi_{2D}\right)}}{h}}\sqrt{\frac{\pi }{2g_{v}n_{s}}}$$ (3)

where *W_m_* is the metal’s WF, *n_s_* is 2D sheet carrier density, *h* is the Planck constant, *g_v_* is the valley degeneracy (*g_v_* = 2 for monolayer vdW materials), *d* is the effective tunneling gap, and *χ*_2*D*_ is the affinity energy of the 2D semiconductor. According to Eq. 3, the theoretical quantum limits of *R_C_* is linearly dependent on the tunneling gap induced by the vdW gap. As depicted in Fig. 5I, the dashed lines that represent *R_c_* at different vdW gaps (*d* = 0 nm, 0.1 nm, 0.2 nm, and 0.3 nm) all display a strong dependency on the channel’s carrier concentration. It implies that, in addition to designing a perfect vdW interface with the matched WF, enhancing the coupling between 2D semiconductor and contact metal is crucial to reduce the *R_c_* further, approaching the quantum limit.

## Driving Current Improvement of 2D Transistors

**Fig. 6.**
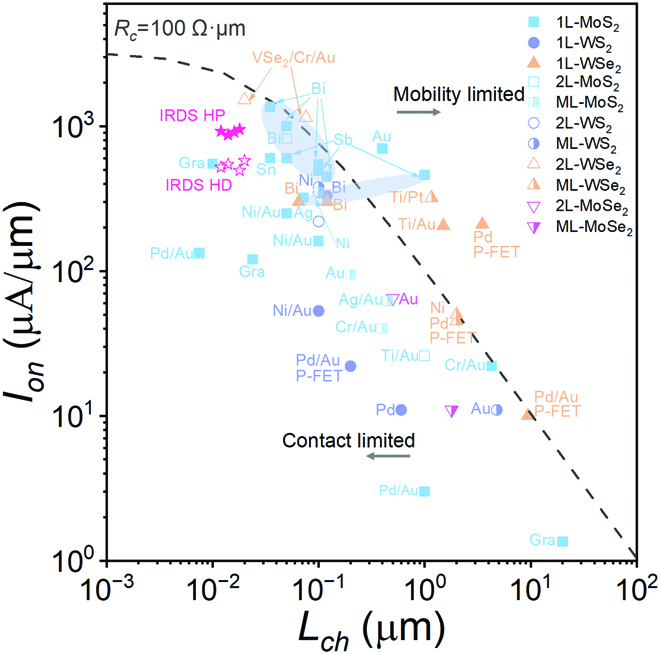
Benchmark of *L_ch_* versus *I_on_* in 2D transistors employing different contact technologies. The gate voltage *V_gs_* is the maximum reported in the respective experimental study. The blue ovals highlight semimetallic contacts such as Bi and Sb. The word “Gra” is the abbreviation of the word “Graphene”. All transistors in the figure are N-FETs except the labeled P-FETs. The *I_on_* values of WSe_2_, MoS_2_, WS_2_, and MoSe_2_ are extracted from Refs. [110, 139, 152, 154, 155, 175–180], Refs. [31, 85, 110, 135, 148, 159, 167, 169, 170, 181–189], Refs. [68, 110, 129, 185, 190–192], and Refs. [156, 193], respectively. HD, high density; HP, high performance.

After going through the optimizations discussed above, we then focus on the most important performance parameter for IC application—*I_on_*, which can be approximated as *I_on_* = *V_ds_*/*R_tot_*. Figure 6 shows the measured *I_on_* as a function of the channel length (*L_ch_*) for monolayer 2D-FETs reported in the literature, where *I_on_* is extracted at *V_D_* equal to 1 V with maximum *V_gs_*. The dashed line illustrates prediction using *R_tot_* = *L_ch_R_sh_* + 2*R_C_*, where the average sheet resistance of the channel *R_sh_* = (*eμn*_2*D*_)^−1^ ≈ 1,524 Ω·sq^−1^ with *n*_2*D*_ = 10^13^ cm^−2^, *μ* = 125 cm^2^ V^−1^ s^−1^ (IRDS 2021), *V_ds_* = 0.75 V (IRDS 2021), and *R_C_* = 100 Ω·μm. Apparently, *I_on_* is significantly limited by the channel mobility for long-channel devices, but becomes strongly dependent on *R_C_* for short-channel devices. As shown in Fig. 6, the theoretical prediction saturates as *L_ch_* < 100 nm. In the past decade, the *I_on_* of short-channel 2D-FET has been successively improved through contact and material optimizations. Nowadays, n-type MoS_2_-FETs demonstrated *I_on_* up to 1,135 μA/μm at *L_ch_* = 35 nm for monolayer [110] and 1,270 μA/μm at *L_ch_* = 50 nm for bilayer [67], respectively. In addition, the p-type 2D-FETs have also achieved several important breakthroughs recently. By modifying the vdW gap at the contact interface and employing platinum electrodes, the contact resistance of p-type few-layer WSe_2_-FET has been reduced to 3.3kΩ·μm [146]. Besides, with a 20-nm-long p-type bilayer WSe_2_-FET, an *I_on_* of 1,720 μA/μm and a contact resistance of 250 Ω·μm have been achieved by employing the vdW–epitaxy VSe_2_ contact [139], showing comparable performance to the n-type counterparts. The red star symbols in Fig. 6 indicate the future requirements for high-performance and high-density devices at technology nodes below 7 nm, respectively. The highest current density in advanced process is about 952 μA/μm at *L_ch_* = 18 nm [9], which means that the device performance of both p-type and n-type 2D semiconductors has to meet or exceed the performance requirements of IRDS. Table summarizes the representative works with detailed metrics of performance, processes, and optimization strategies.

**Table.** The state-of-the-art metrics values in TMD FETs. All values are extracted at *V_ds_* = 1 V and *T* = 300 K. The gate voltage *V_gs_* is the maximum reported in the respective experimental study. The word “Ref.” is the abbreviation of the word “reference”. The table is sorted by material type.

Material	Preparation method	Number of layers	*μ* (cm^2^ V^−1^ s^−1^)	Doping	Contact material	*L_ch_* (μm)	*I_on_* (μA/μm)	*n_2D_* (10^12^ cm^−2^)	*R_C_* (kΩ•μm)	Ref.
MoS_2_	Exfoliated	2	-	N	Ti/Au	1	26	10	-	[31]
MoS_2_	Exfoliated	3	47	N	Cr/Au	0.4	40	37	-	[85]
MoS_2_	Exfoliated	3	-	N	Au	0.2	95	25	2.9	[188]
MoS_2_	Exfoliated	3	50	N	Li phase changed	1.2	85	8	-	[135]
MoS_2_	CVD	1	122.6	N	In/Au	2	18	5	3.3	[147]
MoS_2_	Exfoliated	Few	167	N	In/Au	2	100	3.1	0.8	[147]
MoS_2_	CVD	1	54	N	Au	1	55	-	-	[69]
MoS_2_	CVD	1	102.6	N	Au	0.5	62	-	-	[29]
MoS_2_	CVD	1	30	N	Ti/Au	1.6	10	4	-	[57]
MoS_2_	Exfoliated	1	20	N	Bi/Au	0.035	854	15	0.123	[110]
MoS_2_	CVD	2	122.6	N	Bi/Au	0.05	820	-	0.3	[67]
MoS_2_	Exfoliated	1	-	N	Gra	0.01	185	4.3	5.5	[121]
WSe_2_	CVD	2	-	P	VSe_2_/Cr	0.02	1,520	-	0.25	[139]
WSe_2_	CVD	2	100	P	VSe_2_/Cr	1.8	190	-	-	[194]
WSe_2_	Exfoliated	3	-	N	Au	6.2	12	-	-	[178]
WSe_2_	CVD	1	-	P	Pt	1.5	8	-	229	[146]
WSe_2_	Exfoliated	1	-	N	Bi/Au	0.12	323	-	-	[110]
WSe_2_	Exfoliated	1	180	P	Pd/Au	2.4	34	-	10	[152]
WS_2_	CVD	6	3	P	W	0.04	12	2	-	[68]
WS_2_	Exfoliated	7	50	N	Ni	0.1	380	13	-	[129]
WS_2_	CVD	1	21	N	Bi/Au	0.15	100	-	-	[110]
MoSe_2_	CVD	2	3	N	Au	0.5	65	-	60	[156]

## Summary and Prospect

**Fig. 7.**
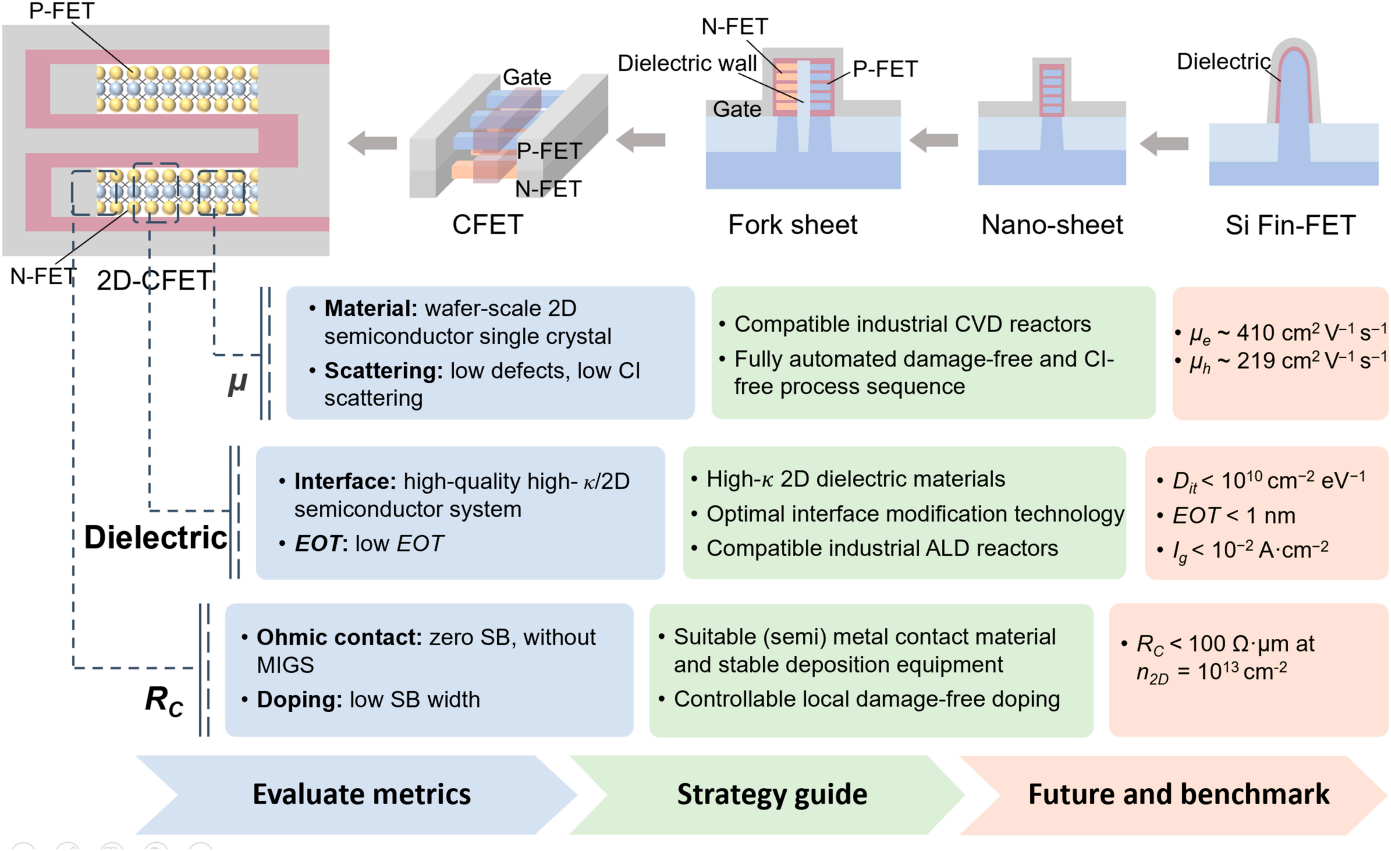
Schematic visualization of transistor development and 2D-FET technology roadmap. The transistor has been structurally innovated step by step, evolving in accordance with Moore's Law and bringing huge benefits to the semiconductor industry. To bring into production, 2D-FET must overcome a series of challenges from laboratory to manufacturing infrastructure, which is outlined in the diagram.

To extend the relentless downscaling, Fin-FET, nanosheet FET, and envisaged Fork-sheet and CFET have been developed (see the top panel of Fig. 7). As the device scale approaches the quantum limit, some issues (e.g., the quantum confinement effect) arise. The advantages of silicon-based technology by means of node evolution, such as energy efficiency and cost, will not exist any longer. Thus, research of new semiconductors suited for industry application is inevitable. 2D-FETs attract tremendous interest for the atomically thin channels to mitigate short-channel effects and to extend “Moore’s Law”. In this review, we summarize the key technologies for achieving high-performance, low-power 2D-FETs through optimizations of mobility, dielectric, and contact. Finally, we show a roadmap of 2D-FET technology in Fig. 7. As for mobility, it is determined by material itself (intrinsic mobility) and impurity scattering as well. The wafer-scale growth of 2D single crystals is highly expected to break through material limitations. Meanwhile, the interface engineering is essential to reduce the number of defects and to restrain the CI scattering. For dielectric engineering, the main aim is to construct a high-quality high-*κ*/2D semiconductor interface and then to realize low *EOT*. The surface modification strategy or the selection of interfacial layer should take low *D_it_*, low *EOT*, and low *I_g_* all into account. For contact engineering, the key is to reduce the height and the width of SB and suppress MIGS, and then to realize stable ohmic contacts with ultralow *R_C_*. Controllable contact-localized doping and suitable contact (semi)metal selection are 2 major strategies. Now, the special semimetal (Sb and Bi) contacts show great potential in this regard [110,148].

The following are several prospects of 2D transistors for future development. Although material-level progresses are the earliest and most accessible, it is still urgent to make a major breakthrough in terms of stable growth method and the relevant industry-compatible CVD reactor for making high-quality 2D semiconductor single crystals over a large area. Due to the good electrostatic control, as well as the smaller bandgap and higher mobility than monolayers, precise layer-controlled TMD single crystal (including bilayer and trilayer) epitaxy with large-area homogeneity would further boost the performance of 2D TMD devices [67]. Also, the synthesis of high-*κ* 2D dielectrics is another vital task that may be easily overlooked. In addition, the present methods to prepare 2D materials mainly adopt solid-phase precursors as raw materials. Their sublimation and diffusion are complex and hard to control, limiting controllability and reproducibility. Thus, the growth method based on all gaseous precursors deserves more exploration.

At the device level, as discussed before, the comprehensive optimization, including mobility, dielectric, and contact, should continue uninterruptedly until the expected target for 2D-FETs is satisfied, i.e., *μ_e_* = 410 cm^2^ V^−1^ s^−1^, *μ_h_* = 219 cm^2^ V^−1^ s^−1^, *R_C_* < 100 Ω·μm at *n*_2*D*_ = 10^13^ cm^−2^, *D_it_* < 10^10^ cm^−2^ eV^−1^, *EOT* < 1 nm, and *I_g_* < 10^−2^ A·cm^-2^, where *μ_e_* (*μ_h_*) is the electron (hole) mobility of n-type (p-type) 2D transistors. Achieving these performance merits actually implies the maturity of 2D-FET technology. From another point of view, the operating frequency of 2D transistors is also a recognized indicator and should be considered to systematically evaluate device performance. At present, the oscillating frequency of the reported ring oscillator based on 2D transistors is still far below 1 GHz, which hardly meets the requirement for commercial ICs. This means that, despite impressive progresses made in laboratory, more effort should be dedicated to device array and integration, i.e., not just a single transistor. Hence, more attention should be paid to their uniformity, reliability, and yield. Moreover, the CFET is the mainstream development direction for 2D transistors. Although the performance of p-type 2D-FET has made great breakthrough, its process compatibility with n-type device remains to be solved.

At the application level, although 2D-FET technology is considered to be one of the most promising candidates in the post-Moore era, it is still implausible that silicon will be fully replaced by 2D semiconductors in the foreseeable future, because of the enormous investment and the immature technology to commercialize 2D transistors. For constant development, some specific “killer application” is necessary. As transferring vdW materials to arbitrary substrates is feasible under room-temperature conditions, monolithic 3D integration of 2D transistors with mainstream semiconductor technologies is a promising way, even being able to substitute the existing amorphous silicon or oxide thin-film transistor technologies. A recent demonstration of fully 3D monolithic, 1270-PPI active-matrix micro-LED displays driven by monolayer MoS_2_ transistors offered a persuasive example [69]. Moreover, due to the excellent flexibility, the low integration temperature, and the outstanding optical transparency of 2D semiconductors, flexible electronics are predictable to become another essential application scenario for 2D transistors. In addition, many potential applications of 2D transistors in sensing, nonvolatile memory, and neuromorphic computing are expected to be achieved soon. Though 2D transistor technology is young, it is very appealing to bring 2D transistors into production by combining continuous process optimization and application exploration.

## Data Availability

All data used in the analysis within this paper and other finding of this study are available from the corresponding author upon reasonable request
